# Komplikationsmanagement von Schaftfrakturen an den oberen Extremitäten: Vom Revisionseingriff bis zur Rehabilitation

**DOI:** 10.1007/s00113-025-01592-9

**Published:** 2025-05-30

**Authors:** Laura A. Hruby, Raffaela Morgenbesser, Florian Wichlas, Alexander Auffarth, Thomas Freude

**Affiliations:** Universitätsklinik für Orthopädie und Traumatologie, Uniklinikum Salzburg, Müllner Hauptstraße 48, 5020 Salzburg, Österreich

**Keywords:** Frakturstabilisierung, Pseudarthrose, Kriegsbedingte Verletzungen, Offene Frakturreposition, Postoperative Versorgung, Fracture fixation internal, Pseudarthrosis, War-related injuries, Open fracture reduction, Postoperative care

## Abstract

Die Behandlung von Schaftfrakturen an der oberen Extremität ist Teil des unfallchirurgischen Klinikalltags. Während bei jungen Erwachsenen sportassoziierte Verletzungen dominieren, steigt bei älteren Patienten die Inzidenz osteoporotischer Frakturen stark an. Trotz etablierter Versorgungsstrategien sind Komplikationen wie Wundheilungsstörungen, Infektionen, neurovaskuläre Verletzungen, Achsen- oder Rotationsfehler, Pseudarthrosen und Implantatversagen häufig. Ein effektives Komplikationsmanagement setzt detaillierte Kenntnisse über morphologische Frakturmerkmale, individuelle Risikofaktoren und differenzierte Revisionsstrategien voraus. Anhand von 2 klinischen Fallbeispielen verdeutlichen wir die Notwendigkeit individuell angepasster Versorgungskonzepte. Aufgrund der steigenden Zahl von zur postprimären Behandlung zutransferierten Kriegsopfern im mitteleuropäischen Raum erhöht sich die Wahrscheinlichkeit, mit ungewohnten Verletzungsmustern, wie etwa nach Explosionen oder Schussverletzungen, konfrontiert zu werden. Dies geht mit neuen Herausforderungen im klinischen Alltag einher. Die physio- und ergotherapeutische Betreuung spielen nach Revisionen von Schaftfrakturen an der oberen Extremität eine zentrale Rolle für das funktionelle Ergebnis. In der geriatrischen Versorgung verbessern alterstraumatologische Konzepte die Mobilisierung und Selbstständigkeit älterer Patienten. Entscheidend bleibt, die Erwartungshaltung der Patienten zu berücksichtigen und die Rehabilitation engmaschig zu begleiten.

## Abstract (en)

The management of shaft fractures of the upper extremities is an integral part of trauma surgery in the clinical routine. While sport-associated injuries predominate in young adults, the incidence of osteoporotic fractures greatly increases in older patients. Despite established treatment strategies, complications such as wound healing disorders, infections, neurovascular injuries, malalignment, pseudarthrosis and implant failure are frequent. An effective complication management requires detailed knowledge of the morphological features of fractures, individual risk factors and differentiated revision strategies. With the presentation of two clinical cases, we aim to highlight the necessity of individualized treatment concepts. Due to the increasing number of war casualties transferred for secondary treatment to Central Europe, the likelihood of encountering unfamiliar injury patterns, such as blast or gunshot wounds, is rising. This development presents new challenges in daily clinical practice. Physiotherapeutic and occupational therapy support play a central role in achieving favorable functional outcomes following revision surgery for diaphyseal fractures of the upper extremities. In geriatric care, orthogeriatric concepts have significantly decreased mortality and improved mobilization as well as self-sufficiency among older patients. Managing patient expectations and providing close supervision throughout the rehabilitation process are decisive factors for successful recovery.

Die konservative und operative Behandlung von Schaftfrakturen an der oberen Extremität sind Teil des unfallchirurgischen Klinikalltags. Während im jungen Erwachsenenalter oft sportassoziierte Verletzungen im Vordergrund stehen, bedingen osteoporotische Frakturen einen starken Anstieg der Inzidenz in geriatrischen Patientenkollektiven.

Trotz etablierter Versorgungsstrategien sind Komplikationen in der Frakturheilung und funktionellen Rehabilitation nach Schaftfrakturen der oberen Extremität keine Seltenheit [3, 5, 9, 11]. Allgemeine chirurgische Risiken beinhalten das Auftreten von Wundheilungsstörungen und Infektionen. Eine neue Herausforderung stellt zudem im Zusammenhang septischer Komplikationen die Keimbesiedelung von komplexen, offenen Schaftfrakturen nach kriegsassoziierten Verletzungen dar. Deren Behandlung hat im mitteleuropäischen Raum in den letzten Jahren durch den Ukraine-Krieg stark zugenommen [4].

Frakturspezifische Komplikationen reichen von der Verletzung neurovaskulärer Strukturen im Zugangsgebiet über repositionsbedingte Achsen- und/oder Rotationsfehler, verzögerte Heilungstendenzen mit Ausbildung von Pseudarthrosen bis hin zum Implantatversagen.

Ein strukturiertes Komplikationsmanagement erfordert sowohl profunde Kenntnisse der morphologischen und biologischen Frakturmerkmale unter Berücksichtigung individueller Risikofaktoren (osteoporotischer Knochen, Co-Morbiditäten, Begleitverletzungen u. v. m.) als auch eine differenzierte Entscheidungsfindung hinsichtlich Revisionsstrategien.

Da allgemeine Aspekte zum Komplikationsmanagement von Humerusschaft- sowie Unterarmfrakturen in den Beiträgen von Sehmisch et al. und Bail et al. zur vorliegenden Ausgabe von *Die Unfallchirurgie* behandelt werden, präsentieren wir in unserer Arbeit 2 klinische Fälle, die die Notwendigkeit patientenspezifischer Versorgungsstrategien verdeutlichen und neue Herausforderungen der modernen Unfallchirurgie beleuchten.

## Pseudarthrose

Bei der osteosynthetischen Versorgung von Unterarmschaftfrakturen sind eine fehlerhafte Reposition und Längenwiederherstellung oder eine ungenügend erzielte Stabilität häufige Ursachen für das Ausbilden einer Pseudarthrose [5, 6]. Neben anhaltenden Schmerzen kann die unzureichende Wiederherstellung anatomischer Längenverhältnisse aufgrund des komplexen biomechanischen Zusammenspiels des proximalen und des distalen Radioulnargelenks sowie der Membrana interossea zu Bewegungseinschränkungen des Ellenbogens und des Handgelenks führen. Die differenzierte bildgebende Untersuchung, mit häufiger Durchführung von 3D-CT-Rekonstruktionen, sowie die Operationsplanung vor Revisionseingriffen sind für den Behandlungserfolg von entscheidender Bedeutung.

### Fallvignette

**Abb. 1:**
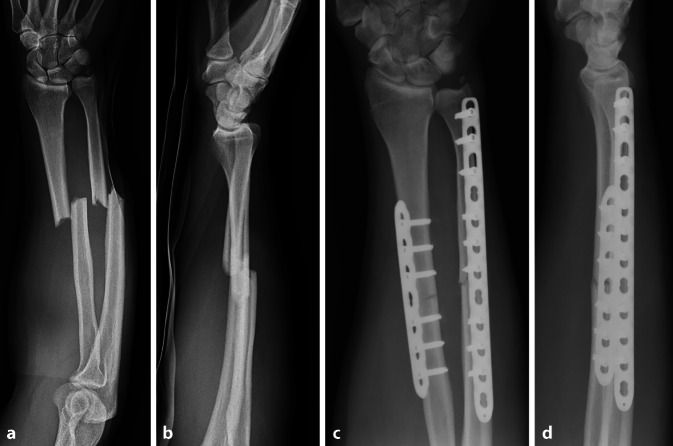
Frakturbilder (**a**, **b**) und primäre osteosynthetische Versorgung (**c**, **d**) einer Unterarmschaftfraktur bei einem 47-Jährigen nach Radsturz

**Abb. 2:**
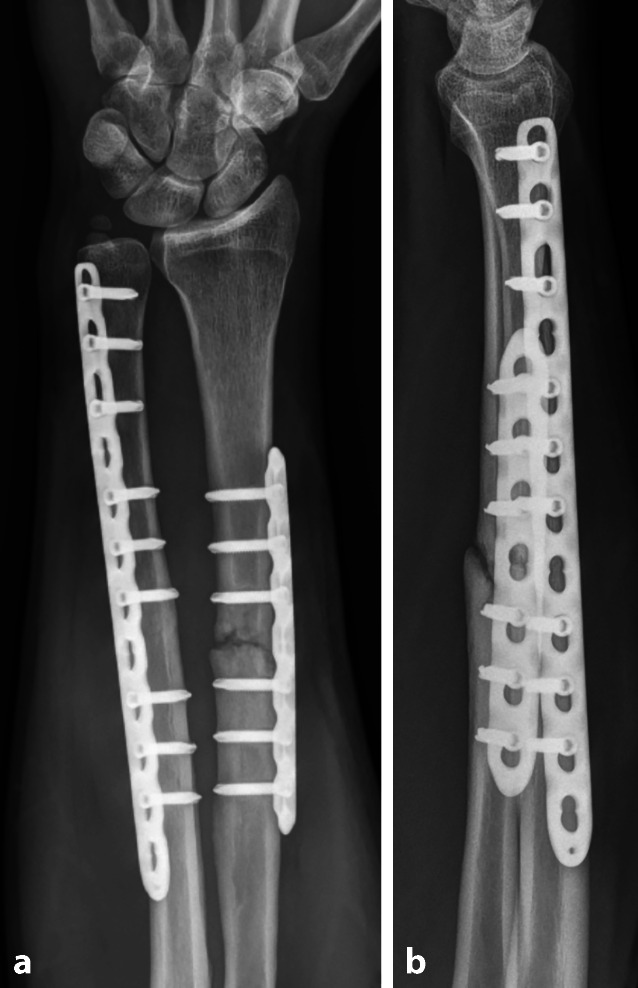
Radiologische Verlaufskontrolle des 47-Jährigen 9 Monate nach osteosynthetisch versorgter Unterarmfraktur. In der a.p. Ansicht (**a**) sowie in der lateralen Ansicht (**b**) zeigt sich eine Pseudarthrose am Radius, die Ulna ist knöchern konsolidiert

**Abb. 3:**
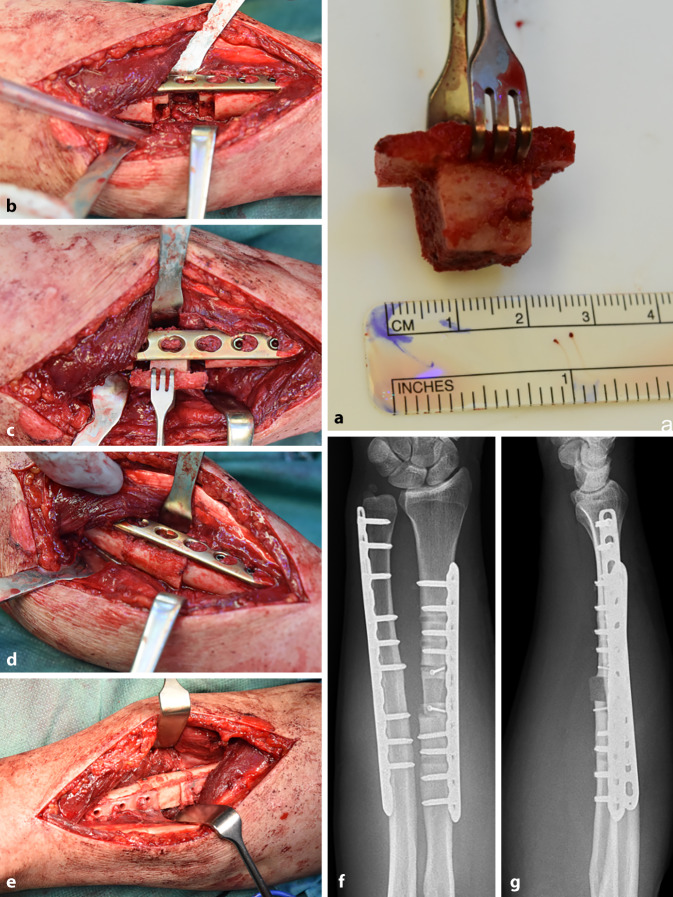
Intra- und postoperative Bilder der Pseudarthrosenexzision und T‑Span-Plastik aus dem Beckenkamm (detaillierte Erklärungen s. Text). (**a**) Aus dem Beckenkamm entnommener und zu einem „T“ modellierter kortikospongiöser Span; (**b**) Pseudarthrosenexzision vor Plattenentfernung; (**c, d**) Einpassung des T-Spans press-fit; (**e**) Fixierung des Spans mittels zweier Kortikalisschrauben; (**f, g**) Post-operatives Röntgenergebnis a.p. und seitlich

Ein 47-jähriger Patient stürzte beim Radfahren und zog sich dabei eine geschlossene Unterarmfraktur im mittleren Drittel zu (Abb. 1a, b). Diese wurde sogleich mithilfe einer winkelstabilen Plattenosteosynthese stabilisiert (Abb. 1c, d). Während die knöcherne Durchbauung an der Ulna zeitlich regelrecht fortschritt, wies der Radius 9 Monate nach oben genanntem Eingriff kaum Zeichen einer Konsolidierung im postoperativen Röntgenbild auf. Es wurde eine Stoßwellenbehandlung initiiert; diese hatte keine ossäre Heilungstendenz zur Folge (Abb. 2). Aus diesem Grund wurde 20 Monate postoperativ eine Revision mithilfe einer Pseudarthrosenexzision und autologen Knochenspanplastik durchgeführt. In der operativen Abfolge wurde die Pseudarthrose im Radius zunächst unter Belassung der primären Platte mit der oszillierenden Säge exzidiert, der entstandene Defekt gemessen und ein trikortikaler Beckenkammspan von 3 cm Länge und 2 cm Höhe entnommen. Dieser wurde sodann entsprechend dem aufnehmenden Defekt zu einem T‑Span am Radius modelliert (Abb. 3a), der Knochen des distalen und proximalen Radius nach entsprechender Markierung zur Passgenauigkeit der Schenkel des T tangential mit der Säge exzidiert (Abb. 3b), der Span schließlich press-fit eingepasst (Abb. 3c, d) und beide Schenkel des T mithilfe von 2 Kortikalisschrauben fixiert (Abb. 3e). Anschließend wurde die alte Platte entfernt und eine 3,5-Locking-Compression-Plate (Fa. DePuy Synthes, Deutschland) eingebracht, wobei der eingebrachte Span über diese mithilfe der exzentrischen Platzierung von frakturnahen Kortikalisschrauben zusätzlich unter Kompression gebracht wurde. Bis auf das unmittelbare, postoperative radiologische Ergebnis (Abb. 3f, g) sind aufgrund des erst kürzlich zurückliegenden Eingriffs keine Verlaufskontrollen verfügbar.

Als Ursachen für die fehlende Stabilität im Frakturbereich des Radius nach dem Primäreingriff können die alleinige Wahl von winkelstabilen Schrauben im Implantat und eine unzureichende Frakturkompression vermutet werden. Die patientenspezifische Modellierung des T‑Spans (in Anlehnung an den J‑Span zur Schulterstabilisierung, der an unserer Abteilung unter Professor Herbert Resch eingeführt wurde [2]), verdeutlicht die Notwendigkeit von interindividuellen Revisionsstrategien im Komplikationsmanagement von Schaftfrakturen der oberen Extremität und erfordert Flexibilität sowie innovatives Denken und Handeln.

## Von der Revision über die Revision zur Revision: Herausforderungen komplexer Kriegsverletzungen

Seit etwa einer Dekade erhöht sich für Unfallchirurg:innen im mitteleuropäischen Raum die Wahrscheinlichkeit, mit ungewohnten Verletzungsmustern, die durch den Einsatz von Hochgeschwindigkeitsschusswaffen oder Explosivstoffen bzw. Sprengsätzen auftreten, konfrontiert zu werden. Dies ist mit einem Anstieg terroristisch motivierter Anschläge und dem Ausbruch des Ukraine-Krieges zu erklären [7].

Nach Explosionsverletzungen mit desaströsen Knochen- und Weichteildefekten sind nicht selten multiple operative Eingriffe notwendig, und es stellen sich im Verlauf immer wieder die Fragen nach der Erhaltungswürdigkeit und -fähigkeit der Extremität sowie nach weiteren Rekonstruktionsoptionen [1]. Zudem kommt erschwerend hinzu, dass solche Verletzungen häufig mit einer Besiedelung multiresistenter Keime einhergehen [4].

### Fallvignette

**Abb. 4:**
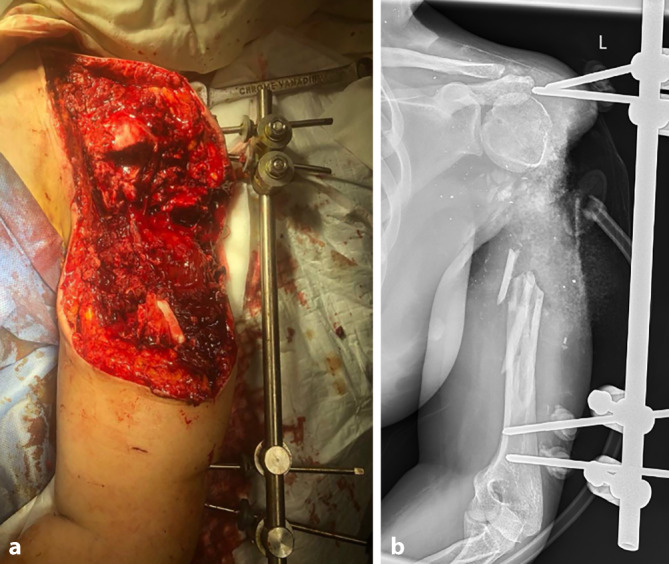
Klinisches Bild und externe Primärversorgung einer 39-jährigen Patientin mittels Fixateur externe (**a, b**), die im Rahmen der kriegerischen Auseinandersetzungen in der Ukraine eine Explosionsverletzung des Oberarms erlitten hatte

Eine 39-jährige Patientin ist im Rahmen einer Explosion in der Ukraine verletzt und dort aufgrund ihrer linksseitigen, offenen Oberarmtrümmerfraktur mit großem Weichteildefekt mithilfe eines Fixateur externe und eines „Vacuum-assisted-closure“(VAC)-Verbands erstversorgt worden (Abb. 4). Drei Wochen später wurde sie zur postprimären Behandlung an unsere Klinik transferiert. Auf eine ebenfalls vorliegende offene Oberschenkeltrümmerfraktur der gleichen Seite wird im vorliegenden Beitrag aufgrund des Leitthemas nicht eingegangen, diese hatte auf die Behandlung und Rehabilitation der oberen Extremität keinen Einfluss.

Im ersten Eingriff wurden der angelegte Fixateur aufgrund der insuffizienten Pin-Lage entfernt, Proben zur bakteriologischen Aufarbeitung eingesandt, die Wunde débridiert, wobei sich eine gute Durchblutung des verbliebenen Oberarmkopfes zeigte, und ein neuerliches VAC-System angebracht. Die Ruhigstellung erfolgte in einer Schulter-Arm-Bandage.

**Abb. 5:**
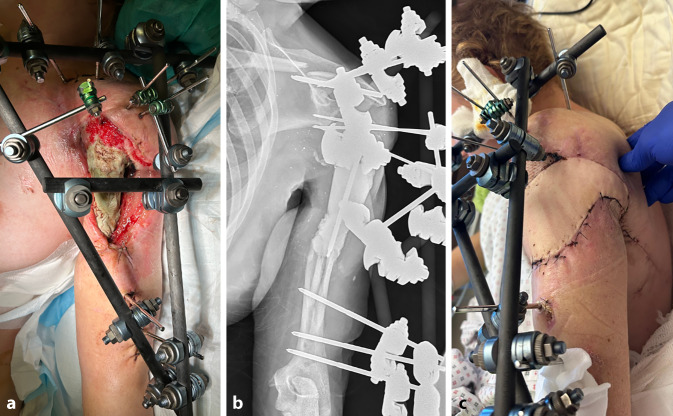
**a** Intraoperatives Bild nach Einbringen des Polymethylmethacrylat(PMMA)-Gentamicin/Vancomycin-Zement-Spacers in den Humerusschaft und Fixateur-Anlage (vor Anlage eines VAC-Verbands). **b** Das postoperative Röntgenbild zeigt die im Verlauf aufgetretene Klavikulafraktur. **c** Zur Weichteildeckung wurden nach mehrfachen VAC-Wechseln und Wundkonditionierung ein Latissimus-dorsi-Lappen und Spalthaut verwendet

Daraufhin wurde zweimalig ein VAC-Wechsel im OP durchgeführt. Sensomotorisch bestand distal des Ellenbogens keinerlei Einschränkung, weshalb die Wunde in einem ausgedehnten Eingriff als Vorbereitung auf einen ossären Rekonstruktionsversuch neuerlich radikal débridiert wurde. Zeitgleich wurden im Sinne der Masquelet-Technik ein Polymethylmethacrylat(PMMA)-Gentamicin/Vancomycin-Zement-Spacer in den Humerusschaft mit zentral einliegendem Schanz-Pin zur Schienung des Konstrukts eingebracht. Ferner wurde ein Fixateur externe angelegt, der den „verrotierten“, freistehenden Humeruskopf auf die Ebene des Glenoids zentrierte und den Oberarm mithilfe von 3 weiteren Pins im distalen Humerusschaft sowie 3 Pins skapulothorakal an die Clavicula und die Spina scapulae fixierte (Abb. 5a, b). Zur weiteren Wundkonditionierung wurde ein VAC-Verband angebracht.

Postoperativ wurde ein Pneumothorax festgestellt; dieser wurde mithilfe einer Bülau-Drainage entlastet. In einer CT-Kontrolle zeigte sich der mediale der beiden Clavicula-Pins als mögliche die Pleura penetrierende Ursache, weshalb dieser zurückgezogen wurde. Als weitere Komplikation trat im Verlauf eine Klavikulafraktur im mittleren Drittel auf Höhe des medialen Pins auf (Abb. 5b), diese wurde aufgrund der geringen Dislokation und der ohnehin bestehenden Transfixation nicht weiter adressiert.

Nach weiteren Wundkonditionierungen wurden schließlich zur plastischen Deckung der Weichteile und des freiliegenden Zement-Spacers von den Kolleg:innen der plastischen Chirurgie ein Latissimus-dorsi Lappen gehoben und zusätzlich Spalthaut transplantiert (Abb. 5c).

Sechs Wochen später erfolgte bei blanden Wundverhältnissen und vitalem Lappen in einem finalen Rekonstruktionsschritt gemeinsam mit den Kolleg:innen der Mund-Kiefer-Gesichtschirurgie (MKG) folgendes Vorgehen: Unsererseits wurde nach Entfernung des PMMA-Zement-Spacers eine überbrückende Plattenosteosynthese mithilfe 10-Loch-PHILOS-Platte (Fa. DePuy Synthes, Deutschland) eingebracht. Vom nun fixierten Humeruskopf wurde zum restlichen Humerusschaft eine Defektstrecke von 13 cm Länge gemessen. Sodann hoben die Kolleg:innen der MKG ein freies osteokutanes Fibulatransplantat von 15 cm Länge mit entsprechender Hautinsel, passend zum entstandenen Weichteildefekt, und mit Anschluss der A. und V. peronaea an die vom distalen Unterarm herauspräparierte und in die Cubita umgeschlagene A. radialis und V. cephalica. Die Fibula wurde distal intramedullär im Humerusschaft positioniert, ebenso proximal im spongiösen Humeruskopf verkeilt und dann mit 2 monokortikalen Schrauben durch die PHILOS-Platte fixiert.

Interdisziplinäre Zusammenarbeit in spezialisierten Boards und Planung gemeinsamer Eingriffe sind essenziell

**Abb. 6:**
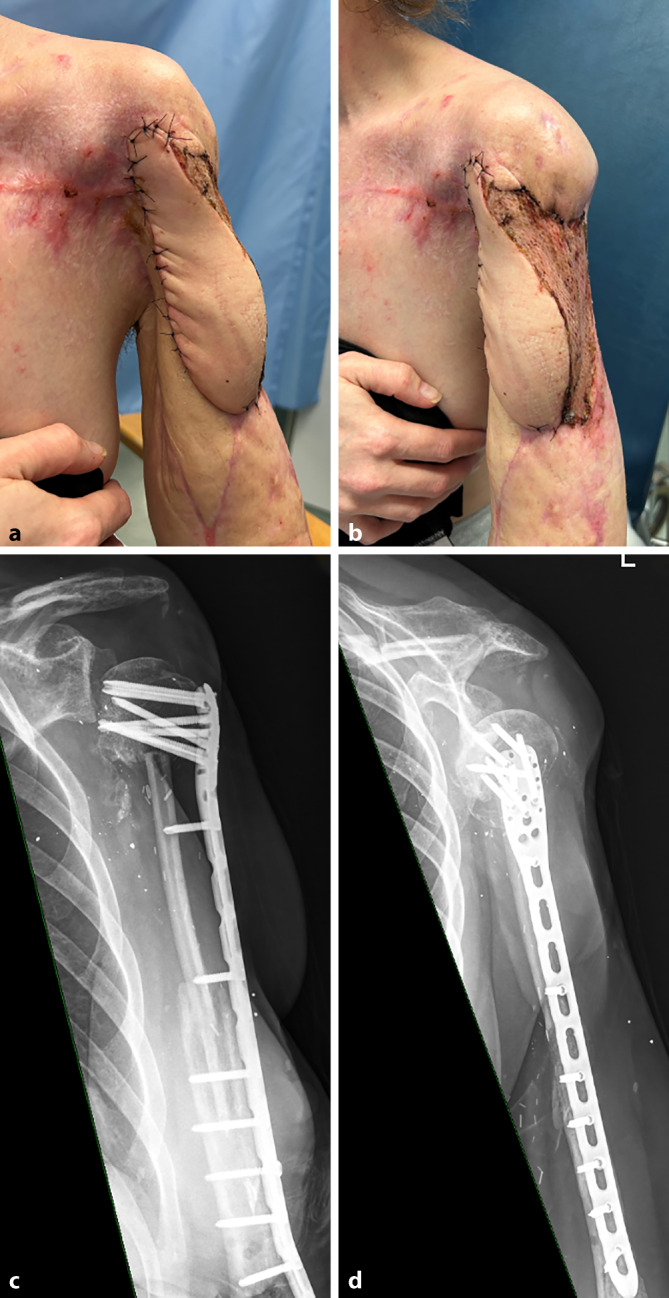
**a**, **b** Neuerliche Lappen- und Spalthautdeckung nach Atrophie der Hautinsel des osteokutanen Fibulalappens über der Platte. **c**, **d** Postoperatives, radiologisches Ergebnis 6 Monate nach freiem Fibulatransfer

Die osteokutane Lappenplastik zeigte sich im Verlauf vital, jedoch die Hautinsel zunehmend atroph, weshalb die Platte bereits wenige Wochen später durch die Haut schimmerte. Nach Fistelbildung, Débridement, Probenentnahme und Bestrahlung der freiliegenden Platte mit einem Erbium-YAG-Laser wurde schließlich von den Kolleg:innen der plastischen Chirurgie neuerlich ein muskulokutaner Lappen transplantiert und der restliche Hautdefekt am lateralen Oberarm mithilfe von Spalthaut gedeckt (Abb. 6a, b). Im Rahmen einer klinisch-radiologischen Verlaufskontrolle 6 Monate nach dem Fibulatransfer (Abb. 6c, d) präsentierte sich die Patientin mit Wackelbewegungen in der Schulter; geschlossenen, blanden Weichteilen sowie einer freien Ellenbogen‑, Handgelenk- und Handbeweglichkeit. Die Hand setzte sie im Alltag ohne Einschränkungen vor und neben dem Körper ein.

Insgesamt waren im Rahmen des Extremitätenerhalts 16 Operationen bei dieser Patienten notwendig, 13 von unfallchirurgisch-orthopädischer Seite, davon eine gemeinsam mit Kolleg:innen der MKG, sowie 3 Eingriffe aus dem Team der plastischen Chirurgie. Die interdisziplinäre Zusammenarbeit, der laufende Austausch über mögliche Revisionsoptionen komplexer Fälle in dafür vorgesehenen Boards sowie die Planung gemeinsamer Eingriffe sind für das funktionelle Outcome solcher Patient:innen von entscheidender Bedeutung.

Genau wie in der prothetischen Versorgung nach der Amputation einer Extremität kann es nach biologischen Rekonstruktionsversuchen vorkommen, dass sich ein objektiv gutes Ergebnis nicht mit dem subjektiven Empfinden des Patienten deckt [8, 10]. Die Entscheidungsfindung muss vordergründig die Wünsche des Betroffenen miteinbeziehen. Die Erwartungshaltung, der Funktionsanspruch und Änderungen dieser subjektiven Empfindungen müssen während des gesamten Behandlungsverlaufes unbedingt hinterfragt, wiederholt ausführlich mit dem Patienten besprochen und ebenso dokumentiert werden. Bei unserem vorgestellten Fall glich sich durch dieses Vorgehen die Erwartungshaltung der Patientin an das zu erreichende Ergebnis an, insbesondere da die Exartikulation im Schultergelenk als mögliche Alternative im Raum stand.

Auf die hochkomplexe Keimsituation dieses Falls kann aufgrund der Größenordnung dieses Beitrags an dieser Stelle nicht eingegangen werden.

## Funktionelle Rehabilitation

Die physio- und ergotherapeutische Begleitung im postoperativen Behandlungsverlauf sind für ein gutes funktionelles Outcome unverzichtbar. Eine enge Zusammenarbeit, die Anbindung von Patient:innen an innerklinische Strukturen sowie der rege Austausch über erfolgte Eingriffe sowie Fortschritte in der Rehabilitation tragen zum Behandlungserfolg bei.

Bei der geriatrischen Traumaversorgung spielt die orthogeriatrische Co-Betreuung eine wichtige Rolle

Insbesondere im höheren Alter ist die Rehabilitation nach Revisionseingriffen zu unterstützen, um ein gutes Ergebnis mit rascher Erlangung einer zufriedenstellenden Funktion zu erzielen. Eine der wichtigsten Entwicklungen im Rahmen geriatrischer Traumaversorgung war die Etablierung von alterstraumatologischen Zentren, i. e. die orthogeriatrische Co-Betreuung von Patient:innen im Alter über 70 Jahren [12], wie sie seit 2013 an unserer Abteilung praktiziert wird. Im Jahr 2021 erfolgte die Zertifizierung zum AltersTraumaZentrum (ATZ) DGU®. Der multidisziplinäre Behandlungsansatz älterer Menschen unter Einbezug von Mitarbeiter:innen der Unfallchirurgie, Geriatrie, Pflege, Physio- und Ergotherapie sowie Sozialarbeit erzielte nicht nur einen Rückgang der Mortalität, sondern auch eine Verbesserung funktioneller Outcomes nach monotraumatischen Ereignissen, wie z. B. der proximalen Femurfraktur [13, 14]. Zum Komplikationsmanagement von Schaftfrakturen der oberen Extremität liegen zwar aktuell keine rezenten Daten vor, die klinische Erfahrung und der Vergleich mit Abteilungen ohne geriatrische Mitbetreuung zeigen jedoch eindeutig auch in diesem Zusammenhang eine raschere Mobilisierung durch gezielte physiotherapeutische Beübung und, damit einhergehend, eine schnellere Wiedererlangung der Selbstständigkeit und einen schnelleren Gebrauch der Extremität im Alltag.

## Fazit für die Praxis

- Das Management von Komplikationen in der Behandlung von Schaftfrakturen bedarf entsprechend ihrer Komplexität interindividueller Versorgungskonzepte, die an die Knochen- und Weichteilsituation, Begleitverletzungen und den Anspruch des Patienten angepasst werden müssen.
- Das effiziente Komplikationsmanagement liegt nicht nur in der lösungsorientierten Behebung eines Problems, sondern v. a. in der Wiederherstellung der funktionellen Integrität der oberen Extremität. Die Erwartungshaltung des Patienten ist vor jedem Eingriff abzuklären und aufklärende Informationen zu postoperativer Rehabilitation und prognostizierter Funktionalität sind bereitzustellen.
- Die physio- und ergotherapeutische Begleitung im postoperativen Behandlungsverlauf ist für ein gutes funktionelles Outcome unverzichtbar.
- Für geriatrische Patient:innen und deren rasche funktionelle Rehabilitation nach Revisionseingriffen an der oberen Extremität ist neben der Zusammenarbeit mit der Physiotherapie die Kooperation mit der Geriatrie entscheidend.
